# Removal of the metronidazole from aqueous solution by heterogeneous electro-Fenton process using nano-Fe_3_O_4_

**DOI:** 10.1016/j.dib.2018.06.118

**Published:** 2018-07-05

**Authors:** Zahra Rahmatinia, Massuomeh Rahmatinia

**Affiliations:** a Department of chemistry, Faculty of Sciences, Ilam University, Ilam, Iran; b Department of Environmental Health Engineering, School of Public Health, Iran University of Medical Sciences, Tehran, Iran

**Keywords:** Metronidazole, Antibiotic, Nano-Fe_3_O_4_, Electro-Fenton

## Abstract

Among drugs, antibiotics have a significant place due to their wide consumption in veterinary and human medicine to prevent and treat microbial infections. In spite of low amounts of antibiotics in the aquatic environments, the repeated incidence of antibiotics has been caused bacterial persistence and adverse effects on health human and aquatic life. Current article evaluated the removal of metronidazole (MNZ) via heterogeneous electro-Fenton (EF) process by nano-Fe_3_O_4_. The response surface methodology (RSM) on Box-Behnken design was applied for modeling and optimization experimental factors such as pH, applied current, and catalyst load. The efficiency of the EF process was maximum (92.26%) under the optimal condition for MNZ removal i.e. 70 mg/L of initial MNZ concentration, pH of 3, 200 mA applied current, 30 min time and 3.2 kWh/m^3^ of energy consumption.

**Specifications Table**

**Table:** 

Subject area	Environmental engineering
More specific subject area	Advanced oxidation process
Type of data	Figure and table
How data was acquired	All degradation tests were done in a reactor 250 mL, equipped with two electrodes graphite- felt (cathode) and platinum sheet (anode).Three level of each variable was evaluated using BOX-Behnken design. The concentration of MNZ was determined by high performance liquid chromatography (HPLC). The characteristic of nano-catalyst was analyzed using field emission scanning electron microscopy (FESEM) (Mira 3-XMU).
Data format	Analyzed
Experimental factors	Measuring of MNZ concentration under various levels of solution pH, catalyst load and applied current to obtain optimal MNZ removal from aqueous solution.
Experimental features	MNZ degradation by EF process using nano-Fe_3_O_4_
Data source location	Iran University of medical sciences, Tehran, Iran
Data accessibility	Data are present in this article only.

**Value of data**

● The nano-Fe_3_O_4_ is reusability and has great stability upon recycling.
● The Box-Behnken design is a useful method to optimize MNZ removal from aqueous solution.
● The obtained data shows heterogeneous EF process by nano-Fe_3_O_4_ an appropriate method for MNZ removal from aqueous solution.

## 1 Data

**Fig. 1:**
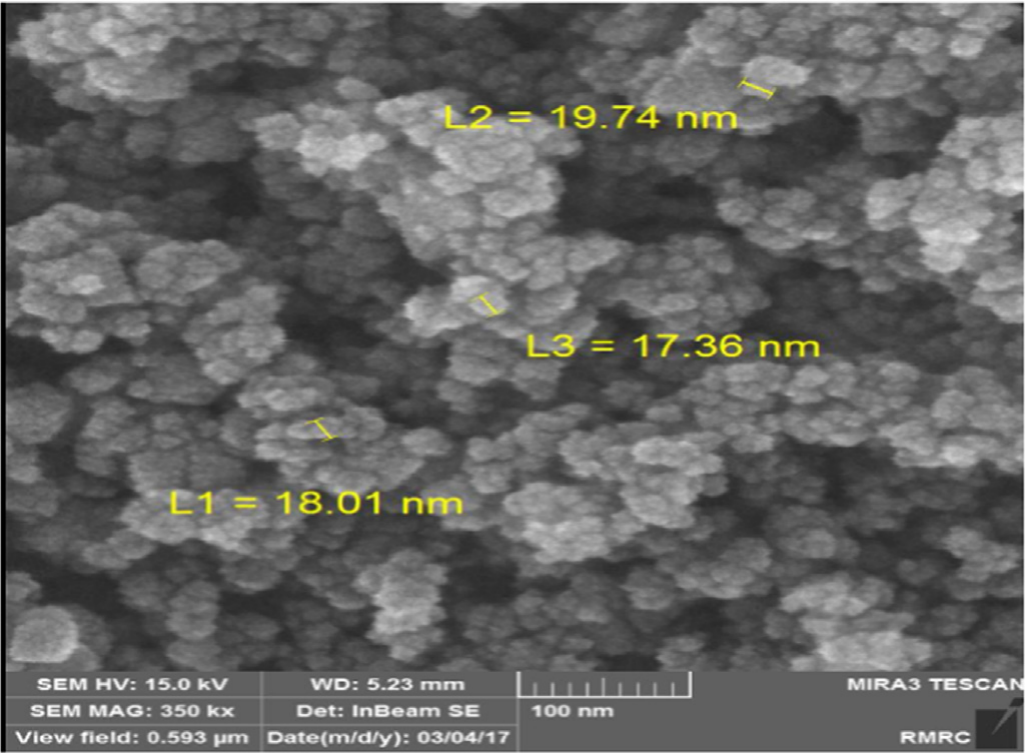
FESEM image of Fe_3_O_4_.

**Fig. 2:**
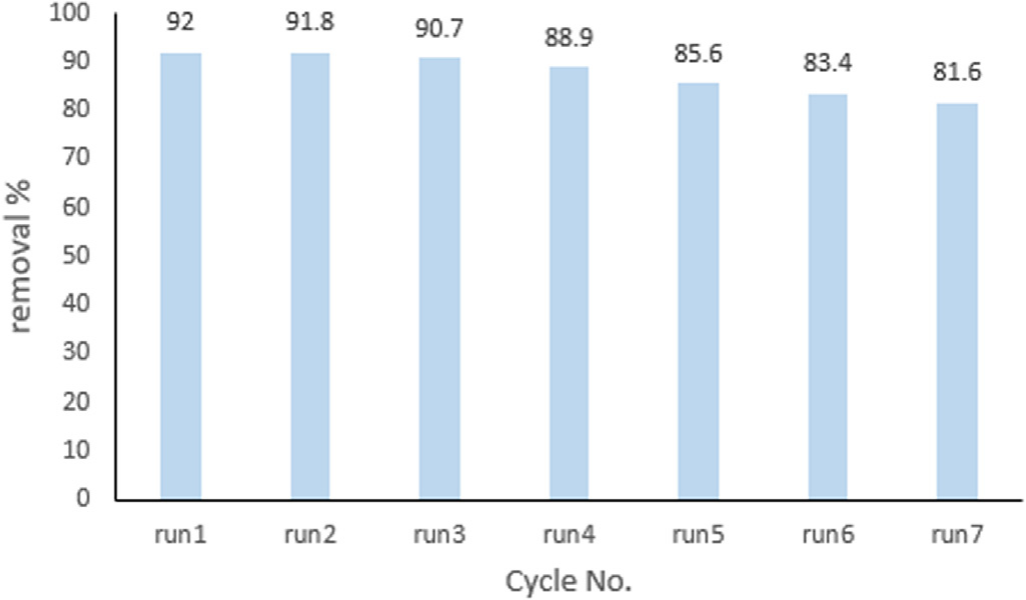
The reusability test of Fe_3_O_4_ catalyst for MNZ degradation during EF process (conditions: Initial MTN concentration: 70 mg/L, applied density: 200 mA, catalyst load: 1 g/L, solution pH: 3, 0.05 M Na_2_SO_4_).

**Fig. 3:**
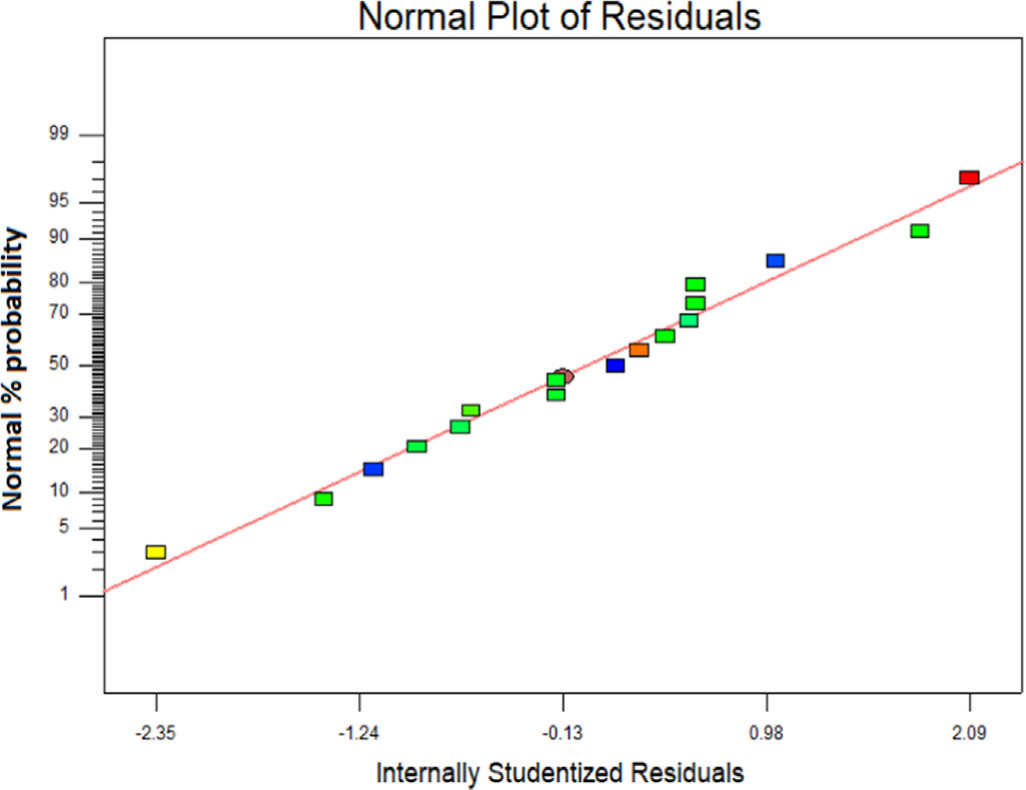
Normal probability plot of studentized residuals.

**Fig. 4:**
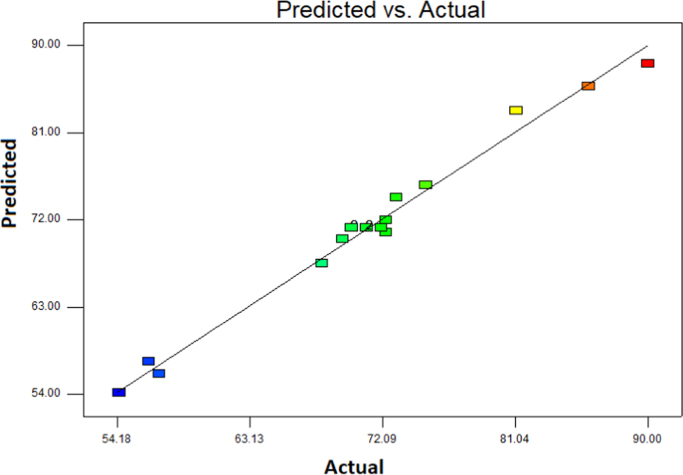
Actual and predicted data of MNZ removal.

**Fig. 5:**
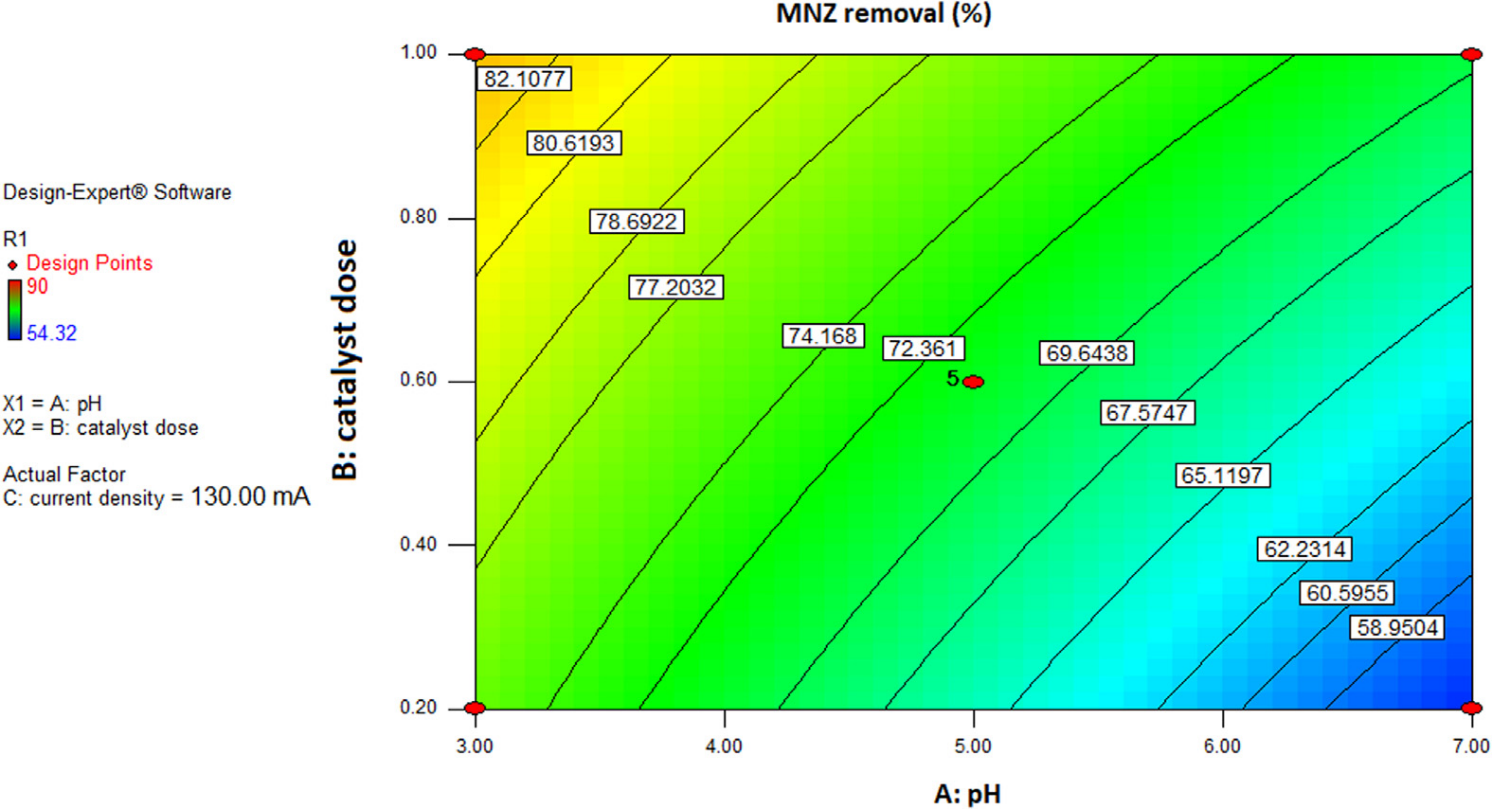
Contour plot for MNZ removal versus pH and catalyst dose by EF process.

**Fig. 6:**
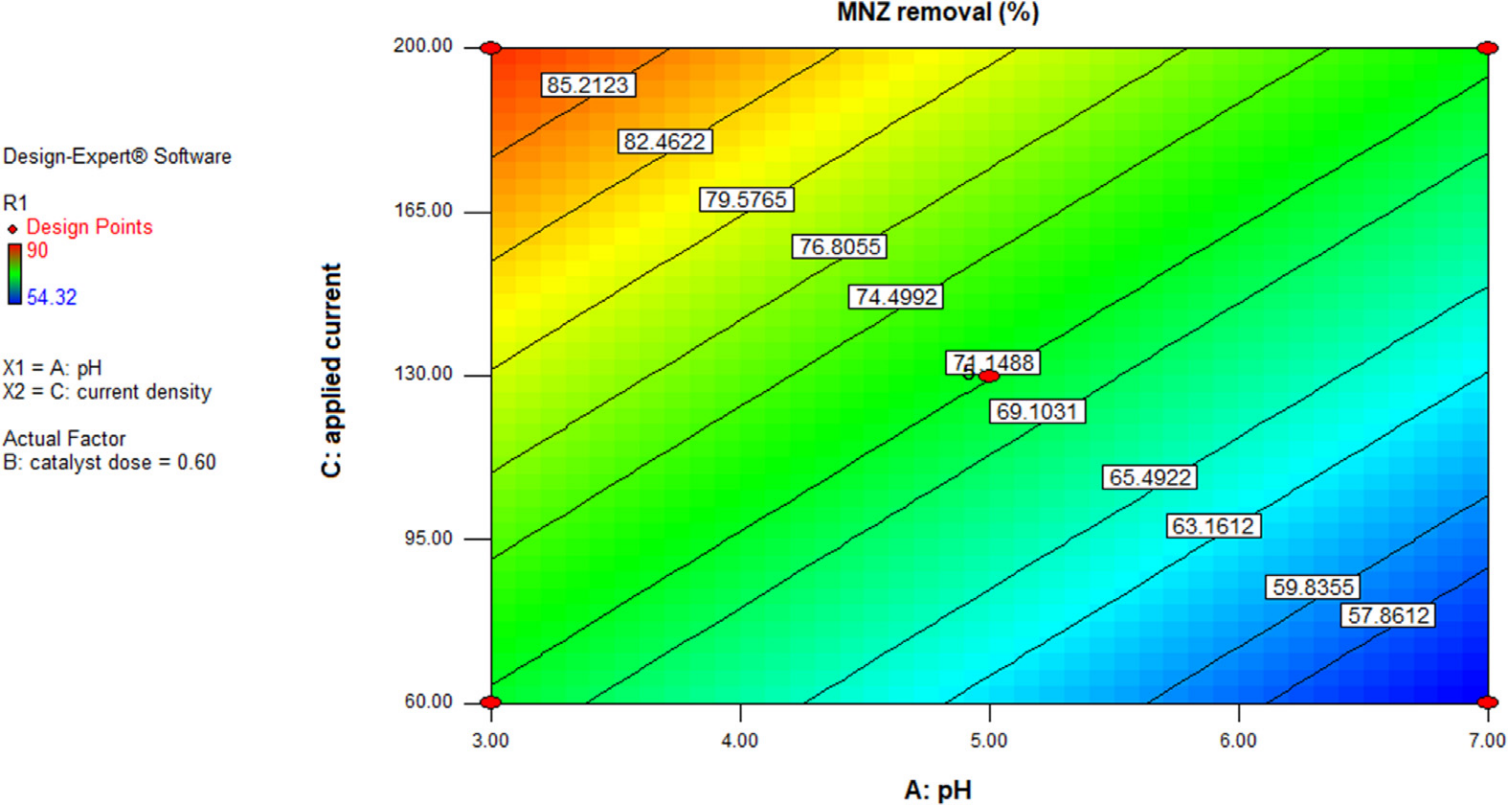
Contour plot for MNZ removal versus pH and applied current by EF process.

**Fig. 7:**
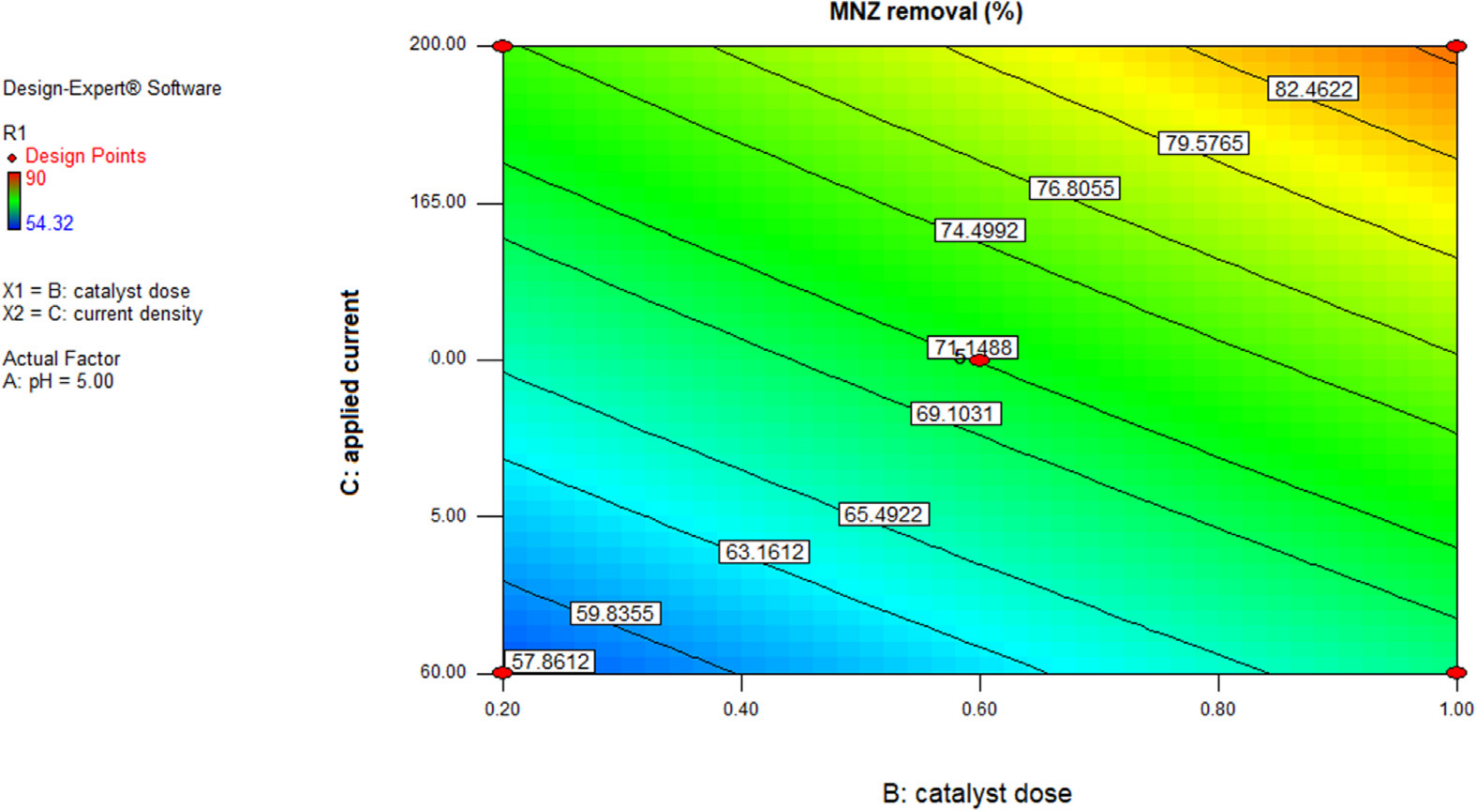
Contour plot for MNZ removal versus catalyst dose and applied current by EF process.

This brief dataset explains the use of EF process using nano-Fe_3_O_4_ for MNZ removal from aqueous solution. Physicochemical characteristics of MNZ are shown in Table 1. Table 2 shows levels of independent variables and experimental range in Box-Behnken design. Box-Behnken design (BBD) was used as a response surface method for optimization of EF process that experimental design and results of MNZ removal have been presented in Table 3. The ANOVA obtained is shown in Table 4 and *P*-value < 0.05 indicate that the model is significant [1], [2]. Also, three variables (initial MNZ concentration, Fe_3_O_4_ dose and pH) were significant terms with *p*-value < 0.05 [3]. The result of FESEM image of Fe_3_O_4_ was presented in Fig. 1. The recyclability of catalyst was evaluated by seven continuous runs and the results are depicted in Fig. 2.The normal probability plot of the studentized Residuals and plot of the predicted versus actual removal values of MNZ are shown in Fig. 3, Fig. 4, respectively. The contour lines plots for the effects of three independent variables on MNZ removal efficiency are shown in Fig. 5, Fig. 6, Fig. 7. A quadratic equation between dependent variable (MNZ removal) and independent variables was obtained according to the Eq. (1).

(1) MNZremoval(%)=71.22–8.17A+5.40B+8.80C+1.57AB+0.070AC+0.32BC

**Table 1:** Physicochemical characteristics of MNZ [4].

Molecular structure	
Molecular formula	C6 H9 N3 O3
Molecular weight (g moL^−1^)	171.15
Melting point (°C)	159–163
Water solubility (g L^−1^)	9.5
pKa	2.55

**Table 2:** Levels of independent variables and experimental range in Box-Behnken design.

Factors	Actual and coded values		
	−1	0	+1
A: pH	3	5	7
B: catalyst dose (g/L)	0.2	0.6	1
C: applied current (mA)	60	130	200

**Table 3:** BBD experimental design and results of MNZ removal.

				Response	
Run	A	B	C	Observed	Predicted
1	7	0.2	130	57	56.1
2	7	1	130	69.4	70.02
3	5	1	200	86	85.74
4	5	0.6	130	71	71.2
5	7	0.6	60	54.32	54.17
6	5	0.6	130	72	71.2
7	5	0.2	200	73	74.29
8	5	0.6	130	71	71.2
9	7	0.6	200	72.3	71.91
10	3	1	130	81.1	83.21
11	5	0.6	130	70	71.21
12	5	0.6	130	72	71.21
13	3	0.2	130	75	75.56
14	3	0.6	60	72.3	70.66
15	5	1	60	68	67.49
16	3	0.6	200	90	88.11
17	5	0.2	60	56.3	57.34

**Table 4:** ANOVA test for quadratic model.

Source	Sum of squares	Degree of freedom	Mean square	*F* value	*P*-value	
					Prob>*F*	
Model	1397.13	6	232.86	127.12	< 0.0001	Significant
A	534.32	1	534.32	291.70	< 0.0001	Significant
B	233.28	1	233.28	127.35	< 0.0001	Significant
C	619.17	1	619.17	338.02	< 0.0001	Significant
AB	9.92	1	9.92	5.42	0.0422	Significant
AC	0.020	1	0.020	0.011	0.9197	
BC	0.42	1	0.42	0.23	0.6414	
Residual	18.32	10	1.83			
Lack of Fit	15.52	6	2.59	3.69	0.1133	Not significant
Pure Error	2.80	4	0.70			
Cor Total	1415.45	16				
R-square	0.9871					
Adj R-square	0.9793					
Pred R-squared	0.9414					
Adequate precision	39.080

## 2 Experimental design, materials and methods

### 2.1 Materials and methods

Nano-Fe_3_O_4_ was made using chemical co-precipitation method [5]. The degradation experiments were carried out in the electrochemical cell (250 mL) that made of Pyrex glass. The anode was platinum sheet with dimensions of 2 cm×1 cm and graphite felt with dimensions 9 cm×9 cm used as cathode. The distance between cathode and anode was 2 cm. DC power source was used to supply the electric current. For each run, 200 mL of MNZ solution (70 mg/L) was introduced in the cell. Na_2_SO_4_ (0.05 M) solution was used as a supporting electrolyte and pH was adjusted by either HCL (0.1 M) or NaOH (0.1 M) solutions. Then the certain amount of nano-catalyst was added, and solutions with a magnetic bar were stirred. Compressed air was bubbled into the solution at 1 L min^−1^, starting 20 min before electrolysis. Finally, MNZ samples were taken at contact time of 30 min for measuring MNZ removal by high performance liquid chromatography (HPLC, CE4200-cecil, England) at 318 nm. The equation below was used for obtaining the removal efficiency (ƞ %) as follows:

(2) $$\left(\frac{C_{0}-C_{F}}{C_{0}}\right)\times 100\%$$

Where, C_0_ is the initial concentration and C_t_ is residual concentration of MNZ [6], [7]. Also, electrical energy consumption (P (kWh/m^3^)) was calculated using Eq. (3) as follows:

(3) $$E=\frac{Eit}{V}$$

Where, E is the cell voltage (v), i is the applied current (A), t is the electrolysis time (h), and v is the volume of the solution (m^3^) [8].

### 2.2 Experimental design

#### 2.2.1 Box-Behnken design experiments

The experiments designed by Design-Expert software (version 7), based on Box*–*Behnken design (BBD) and total experiments were 17 runs. BOX-Behnken design was used to analyze three parameters i.e. pH (3–7), catalyst dose (0.2–1 g/L) and applied current (60–200 mA) on MNZ removal efficiency and removal optimum conditions.
